# Cysteinyl Leukotriene Pathway and Cancer

**DOI:** 10.3390/ijms23010120

**Published:** 2021-12-23

**Authors:** Ming-Ju Tsai, Wei-An Chang, Cheng-Hao Chuang, Kuan-Li Wu, Chih-Hung Cheng, Chau-Chyun Sheu, Ya-Ling Hsu, Jen-Yu Hung

**Affiliations:** 1 Division of Pulmonary and Critical Care Medicine, Department of Internal Medicine, Kaohsiung Medical University Hospital, Kaohsiung Medical University, Kaohsiung 807, Taiwan; SiegfriedTsai@gmail.com (M.-J.T.); 960215kmuh@gmail.com (W.-A.C.); aeafish@gmail.com (C.-H.C.); 980448kmuh@gmail.com (K.-L.W.); Markbruse617@gmail.com (C.-H.C.); sheucc@gmail.com (C.-C.S.); 2 School of Medicine, College of Medicine, Kaohsiung Medical University, Kaohsiung 807, Taiwan; 3 Department of Respiratory Care, College of Medicine, Kaohsiung Medical University, Kaohsiung 807, Taiwan; 4 Graduate Institute of Medicine, College of Medicine, Kaohsiung Medical University, Kaohsiung 807, Taiwan; yainghsu@kmu.edu.tw; 5 Department of Internal Medicine, Kaohsiung Municipal Ta-Tung Hospital, Kaohsiung Medical University, Kaohsiung 807, Taiwan

**Keywords:** leukotriene, montelukast, zafirlukast, chemoprevention, cell death, apoptosis, CysLT_1_

## Abstract

Cancer remains a leading cause of death worldwide, despite many advances being made in recent decades. Changes in the tumor microenvironment, including dysregulated immunity, may contribute to carcinogenesis and cancer progression. The cysteinyl leukotriene (CysLT) pathway is involved in several signal pathways, having various functions in different tissues. We summarized major findings of studies about the roles of the CysLT pathway in cancer. Many in vitro studies suggested the roles of CysLTs in cell survival/proliferation via CysLT_1_ receptor (CysLT_1_R). CysLT_1_R antagonism decreased cell vitality and induced cell death in several types of cancer cells, such as colorectal, urological, breast, lung and neurological malignancies. CysLTs were also associated with multidrug resistance of cancer, and CysLT_1_R antagonism might reverse chemoresistance. Some animal studies demonstrated the beneficial effects of CysLT_1_R antagonist in inhibiting tumorigenesis and progression of some cancer types, particularly colorectal cancer and lung cancer. The expression of CysLT_1_R was shown in various cancer tissues, particularly colorectal cancer and urological malignancies, and higher expression was associated with a poorer prognosis. The chemo-preventive effects of CysLT_1_R antagonists were demonstrated in two large retrospective cohort studies. In summary, the roles of the CysLT pathway in cancer have been delineated, whereas further studies are still warranted.

## 1. Unmet Need of Cancer Treatment

Cancer is a major public health issue/concern worldwide and is the leading cause of death in many countries, including Taiwan [1,2]. Many types of procedures and medications are now available for cancer treatment, including surgery, radiotherapy, chemotherapeutic agents and targeted therapy. The development of immunotherapy seems to open a new era of cancer treatment. Check point inhibitor, one of the different types of immunotherapeutic agents, has been demonstrated to treat certain types of cancer alone or in combination with chemotherapeutic agents [3]. Although many anti-cancer treatments are available, drug resistance usually develops sooner or later, and some patients had poor or even no response to the treatment at the beginning. As a result, high cancer mortality remains, suggesting the urgent need of a large improvement in cancer treatment.

The tumor microenvironment is composed of the extracellular matrix and basement membrane, endothelial cells, cancer-associated fibroblasts, neuroendocrine cells, adipose cells, pericytes and tumor-infiltrating immune cells [4]. A tumor and its microenvironment interact constantly and influence each other. Such interactions begin at the very early phase of tumor formation, and continue during primary growth, local invasion, intravasation and establishment at the metastatic site. The role of the tumor microenvironment has been demonstrated to modulate the aggressiveness, motility, dissemination and colonization of cancer cells in distal organs in the past two decades [5]. Inflammation, including the leukotriene pathway, has been proposed as one of the mechanisms in tumor initiation, progression and metastasis [6]. Cancer-associated immune dysregulation is also a key contributor for tumor progression and metastasis.

By understanding the role of tumor microenvironment and cancer immunology in cancer progression, as well as the underlying detailed molecular mechanisms, we will have a better chance of developing novel diagnostic modalities and therapeutic agents for cancer diagnosis and treatment.

## 2. Cysteinyl Leukotriene Pathway

### 2.1. Leukotrienes

Leukotrienes were initially identified in the late 1970s, as a family of inflammatory lipid mediators synthesized from arachidonic acid (AA) in different cells, including mast cells, eosinophils, neutrophils, basophils and macrophages [7].

**Figure 1:**
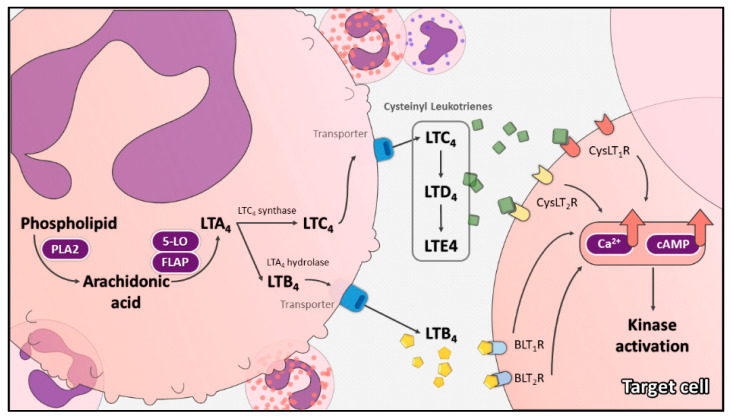
The cysteinyl leukotriene pathway. Leukotriene A_4_ (LTA_4_), originated from arachidonic acid, is converted into leukotriene C_4_ (LTC_4_) by LTC_4_ synthase. After transporting into the extracellular milieu, LTC_4_ is converted to leukotriene D_4_ (LTD_4_) and then leukotriene E_4_ (LTE_4_). The cysteinyl leukotrienes, including LTC_4_, LTD_4_ and LTE_4_, bind to cysteinyl leukotriene receptors, mainly CysLT_1_R and CysLT_2_R, to activate signaling pathways in the target cells.

While the cell membrane encounters stimulation or injury, phospholipase A2 catalyzes the hydrolysis of AA from phospholipids of cell membrane (Figure 1). The stimulation could be inflammatory or immunologic processes, such as immediate hypersensitivity, platelet activating factor or calcium ionophore. The liberated free AA will then be dehydrated by 5-lipoxygenase (5-LO) in concert with 5-lipoxygenase-activating protein (FLAP) and become unstable epoxide leukotriene A_4_ (LTA_4_) [8,9]. In the next step, the unstable epoxide LTA_4_ is converted by LTA_4_ hydrolase into leukotriene B_4_ (LTB_4_) and by leukotriene C_4_ (LTC_4_) synthase into LTC_4_ [10]. After transporting into the extracellular milieu, LTC_4_ will be converted to leukotriene D_4_ (LTD_4_) [11]. Finally, dipeptidase deprives LTD_4_’s glycine residue, turning LTD_4_ into leukotriene E_4_ (LTE_4_) [12].

Due to lacking the peptide side chain of cysteinyl leukotrienes, LTB_4_ was not classified as cysteinyl leukotriene [13]. As a classical chemoattractant, LTB_4_ could trigger aggregation and adherence of leukocytes to the endothelium. Furthermore, it also regulates the immune responses associated with host-defense against infections. LTB_4_ is involved in many inflammatory diseases, including dermatitis, arthritis, nephritis and chronic obstructive pulmonary disease [10].

### 2.2. Cysteinyl Leukotrienes

The cysteinyl leukotrienes (CysLTs) include LTC_4_, LTD_4_ and LTE_4_ (Figure 1). Among them, LTE_4_ is the most stable, which can be measured in the urine. The urinary LTE_4_ level could therefore be used as a marker of ‘whole body’ leukotriene synthesis [14]. LTC_4_ is an 18-kDa membrane protein. It is a noncovalent homodimer. Mg^2+^ could augment the enzyme activity of LTC_4_; on the other hand, Co^2+^ and the function of FLAP inhibitor MK886 could inhibit it [15]. LTD_4_ increased cytosolic calcium and activated the MAPK pathway in THP-1 cells, a human monocytic leukemia cell line [16,17]. Activation of PKC inhibited LTC_4_ synthase activity and attenuates production of CysLTs in an eosinophilic sub-strain of human myeloid leukemia cell line HL-60 [18]. CysLTs are well-known for their role in inflammation, and have been reported to have pro-angiogenic activities [19,20,21].

There are three groups of cysteinyl leukotriene receptors (CysLTRs), including CysLT_1_R, CysLT_2_R and CysLT_3_R. The affinity of CysLTs to their receptors are different:

the affinity of CysLTs to CysLT_1_R: LTD_4_ > LTC_4_ >> LTE_4_;
the affinity of CysLTs to CysLT_2_R: LTD_4_ = LTC_4_ >> LTE_4_;
the affinity of CysLTs to CysLT_3_R: LTC_4_ > LTD_4_ [22].

### 2.3. CysLT_1_ Receptor (CysLT_1_R)

CysLT_1_R distributes in various human tissues, including the respiratory system, peripheral blood leukocytes, gastrointestinal system and brain [7]. Smooth muscle cells, epithelial cells, interstitial lung macrophages and basophils accumulating in the airways of asthma patients could express CysLT_1_R [7,23,24]. The expression of CysLT_1_R could also be found in peripheral blood leukocytes, including monocytes, macrophages, eosinophils, pregranulocytic CD34^+^ cells, neutrophils and some B lymphocytes [7]. Tumors of the colon and brain also express CysLT_1_R [22].

CysLT_1_R is involved in several signal pathways. LTD_4_ is able to induce elevation of intracellular free Ca^2+^ concentration and phosphatidylinositol metabolism [25]. Via non-voltage gated channels, LTD_4_ could lead to constriction of the small bronchioles [26]. Activating CysLT_1_R induces phosphorylation of mitogen-activated protein kinase (MAPK) through a G_i/o_-protein in mesangial cells, airway smooth muscle cells and human mast cells [22].

STAT-1 was demonstrated to be involved in the signal transduction mechanism of the CysLT_1_R; phosphorylation of STAT-1, through protein kinase C (PKC) and ERK1/2 activation, causes expression of ICAM-1 and increased eosinophil adhesion [24]. In studies of human asthma smooth muscle cells, LTD_4_ stimulation of G_i/o_-coupled CysLT_1_Rs leads to the transactivation of the epidermal growth factor receptors (EGFRs) through the intervention of PI3K and ROS, followed by the classical Ras-ERK1/2 signaling pathway. It could then control G1 progression and cell proliferation [23]. CysLT_1_R also plays an important role in Alzheimer’s disease. In the study of primary cultured neurons, LTD_4_ was demonstrated to cause the production of Aβ by enhancement of β- or γ-secretase resulting from activating the CysLT_1_R-mediated NF-κB signaling pathway [27].

### 2.4. CysLT_2_ Receptor (CysLT_2_R)

The mRNA of CysLT_2_R mRNA expression is high in the heart, interstitial macrophages of lung, brain, spleen, lymph nodes and peripheral blood [28]. The expression level of CysLT_2_R mRNA is particularly high in the hypothalamus, thalamus, putamen, pituitary gland and medulla of brain. In the peripheral blood, eosinophils particularly express very high level of CysLT_2_R [28]. On the cell surface of human umbilical vein endothelial cells, CysLT_2_R is highly expressed and might be involved in leukotriene-dependent vascular reactions [29].

CysLT_2_R induced microglia M1 polarization through activating the NF-κB pathway, and might promote inflammation and neuronal damage [30]. Aspirin-exacerbated respiratory disease is associated with idiosyncratic CysLT- and mast cells-driven reactions to aspirin. In aspirin-exacerbated respiratory disease, CysLT_2_R signaling on platelets could use RAGE/HMGB1 as a link to the downstream type 2 respiratory immunopathology and IL-33-dependent mast cell activation typical of aspirin-exacerbated respiratory disease [31].

### 2.5. CysLT_3_ Receptor (CysLT_3_R)

CysLT_3_R has high-affinity for LTE_4_ [32]. CysLT_3_R was demonstrated to be expressed on murine airway epithelial cells and to mediate goblet cell mucin release in response to exogenous LTE_4_ [33]. CysLT_3_R could also regulate the numbers of goblet cells in the nasal mucosa. The aeroallergen and LTE_4_-elicited CysLT_3_R-dependent type 2 lung inflammation could be attenuated by blocking IL-25. Therefore, CysLT_3_R may have the potential to be a therapeutic target for inflammatory lung disease [34].

### 2.6. Cysteinyl Leukotriene Pathway Antagonists

In daily clinical practice, medications targeting the CysLT pathway are implicated in the treatment of asthma and allergic rhinitis. In patients with asthma, a chronic inflammatory lung disease characterized by airway hyperreactivity, CysLTs increased vascular permeability and smooth-muscle contraction, which are the causes of patients’ symptoms [35]. The pharmacological mechanism of montelukast, zafirlukast and pranlukast in treating asthma is antagonism of CysLT_1_Rs (Table 1). The other drug, zileuton, can be used for treating asthma by inhibiting 5-LO. However, the responses to these pharmacologic blockades remain heterogenous [35].

## 3. In Vitro Studies about the Roles of Cysteinyl Leukotriene Pathway in Cancer

Major findings of in vitro studies about the roles of the CysLT pathway in cancer are summarized in Table A1 in Appendix A.

### 3.1. Hematologic Malignancies

Studies about physiological roles of CysLTs in cancer cells have been initiated since 1980s, mainly in leukemia cells [36,37]. Increased LTC_4_ production was noted in chronic myeloid leukemia (CML) [37,38]. Some early studies using leukemia cell lines further enriched our knowledge about the role of CysLTs in cell biology [16,17,18].

In line with clinical practice, tyrosine kinase inhibitors, such as imatinib, dasatinib and nilotinib, inhibited cell growth of CML cells, and montelukast further reduced cell proliferation in a dose-dependent manner as key proteins of the leukotriene pathway were expressed in these CML cells [39]. A subsequent study revealed that montelukast, through a CysLT_1_R-dependent pathway, induced apoptosis of CML cells by inducing Bax overexpression, cytochrome C release, PARP-1 cleavage and caspase-3 activation, which could be additive to the pro-apoptotic effect of imatinib; montelukast also altered Wnt/β-catenin signaling, inducing phosphorylation of β-catenin and downregulating the downstream target c-myc [40].

In terms of chronic lymphocytic leukemia (CLL), two CLL cell lines (EHEB and MEC-1 cells) expressed high levels of CysLT_1_R and low level of CysLT_2_R [41]. LTD_4_ induced CysLT_1_R-mediated calcium fluxes, actin polymerization, chemotaxis and activation of MAPK pathway in CLL cells [41]. CysLT_1_R antagonists (MK571 and LY171883) reduced viability and increased apoptosis of CLL cells [41].

CysLT_1_R expression was demonstrated in human primary mediastinal B-cell lymphoma cell lines (Med-B1, Karpas-1106P); CysLTs induced a calcium signal in Med-B1 cells, which could be blocked by zafirlukast [42]. Some of the human Hodgkin lymphoma cell lines, including L1236 and KMH2 cells, expressed functional CysLT_1_R, responding with a robust calcium signal upon LTD_4_ challenge, which could be blocked by zafirlukast [43].

### 3.2. Colorectal Cancer

The role of the CysLT pathway has been extensively studied in intestinal epithelium and associated tumors. The expression of CysLT_1_R was demonstrated in intestine 407 (Int 407), a non-transformed human embryonic intestinal epithelial cell line and two human colon cancer cell lines, Caco-2 and SW-480 [44].

Several studies have demonstrated the role of the CysLT pathway in cell survival of colorectal cancer cells. Overexpression of CysLT_1_R increased cell viability in Caco-2 cells [44]. A study using Int 407 found that LTD_4_ was capable of preventing apoptosis induced by NS-398, a COX-2 inhibitor [45]. Further study revealed that LTD_4_ not only reversed the apoptosis induced by COX-2 inhibition but also reduced the apoptotic potential by lowering the basal level of caspase 8 activation and truncated Bid generation [46]. LTD_4_ enhanced proliferation via a distinct Ras-independent, PKCɛ-dependent activation of Erk-1/2 and a parallel Ras-dependent signaling pathway [47]. A study using Int 407 and Caco-2 cells demonstrated that LTD_4_ induced upregulation of COX-2 and Bcl-2 through a pertussis toxin sensitive G-protein and MAPK pathway [48]. In both Int 407 and Caco-2 cells, LTD_4_ stimulation induced activation and nuclear translocation of cytosolic phospholipase A_2_α, an important regulator of colon tumor growth, via a calcium-dependent mechanism involving activation of PKC and the MAPK pathway [49]. LTD_4_ increased the level of free β-catenin in Int 407 cells; the increased free β-catenin translocated to the nucleus where it activated TCF/LEF transcription factors; the increased free β-catenin also translocated to the mitochondria where it associated to the antiapoptotic protein Bcl-2 [50]. Further studies showed that LTD_4_ increased mitochondrial metabolic activity and gene transcription and increased reactive oxygen species levels and subsequent activations of the p65 subunit of NF-κB, presumably through β-catenin accumulation in the mitochondria [51]. Collectively, these findings lend credence to the idea that the CysLT pathway potentially provides intrinsic oncogenic signals involving cell survival and anti-apoptosis.

Furthermore, LTD_4_ increased the motility of Int 407 cells via a PI3K/Rac signaling pathway [52]. LTD_4_ increased β-catenin level in colon cancer cells [53]. LTD_4_ induced nuclear translocation of β-catenin, upregulated β-catenin target genes and increased the proliferation and migration in HCT116 cells, but not in HT29 cells; the effect could be prevented by pretreatment with ZM198,615, a CysLT_1_R antagonist [53].

CysLT_1_Rs were found in the plasma membrane and outer nuclear membrane in both Int 407 and Caco-2 cells [54]. The colorectal carcinoma cell line, Caco-2 cells, appeared to have greater intracellular formation of CysLTs and more CysLT_1_Rs in both plasma membrane and outer nuclear membrane than the non-tumor cell line, Int 407 cells [54]. Another study found that the basal level of CysLT_1_R was higher in several colon cancer cell lines (HT-29, SW-480, Caco-2 and HCT-116) compared to Int 407 cells [55]. LTD_4_ significantly increased CysLT_1_R expression in Int 407 cells, but not in colon cancer cell lines; LTD_4_ induced upregulation of CysLT_2_R in several colon cancer cell lines [55]. LTD_4_ could also induce nuclear translocation of CysLT_1_Rs from the plasma membrane to the nucleus in Int 407 cells, but not in Caco-2 cells [54].

A study using several intestinal epithelial cell lines found the autocrine pattern of endogenously produced CysLTs which mediated the survival and proliferation of intestinal epithelial cells via CysLT_1_R signaling [56]. MK571, a CysLT_1_R antagonist, induced apoptosis and dose-dependent proliferation inhibition in two non-tumor cell lines (Int 407 and IEC-6 cells), but only led to proliferation reduction without apoptosis in the tumor intestinal cell lines (Caco-2, SW480, HCT-116 and HT-29 cells); the presence of nuclear CysLT_1_Rs in intestinal cancer cells, which are inaccessible to the receptor antagonist, might provide a clue to the finding [56]. In a study with HT-29 and SW-480, montelukast prevented LTD_4_-induced colony formation and disrupted colonospheres as well as downregulation of cancer stem cell markers (ALDH1 and DCLK1), suggesting the beneficial effect in minimizing cancer stem cells of CysLT_1_R inhibition [57]. In addition, 1,4-dihydroxy quininib reduced clonal formation and gene silencing of CysLT_1_R significantly reduced expression of angiogenic marker calpain-2, which further confirmed the importance of CysLT_1_R in cancer progression and angiogenesis [58].

The expression of low-affinity CysLT_2_R, compared with CysLT_1_R, was higher in a non-tumor cell line (Int 407) but was lower in two colon cancer cell lines (Caco-2 and SW480) [59]. Similar to CysLT_1_R, CysLT_2_R was found to be located both at the plasma membrane and the nuclear membrane [59]. Although CysLT_2_R signaling had no effect on cell proliferation or apoptosis of Caco-2 cells, LTC_4_ increased the activity of alkaline phosphatase and aminopeptidase N, suggesting the role of CysLT_2_R in cellular differentiation [59]. A subsequent study showed that IFN-α could upregulate CysLT_2_R in Caco-2 cells [60]. LTC_4_ induced expression of mucin-2, and the effect could be blocked by AP 100984 (a specific CysLT_2_R antagonist) but not by montelukast (a specific CysLT_1_R antagonist) [60]. CysLT_2_R signaling was able to suppress cell migration induced by epidermal growth factor (EGF) signaling in Int 407 cells [60]. All-trans retinoic acid (ATRA) treatment increased CysLT_2_R expression without affecting CysLT_1_R level, and upregulated LTC_4_ synthase in SW480 cells; the effect was not observed in HCT-116 cells, which was ATRA-resistant [61]. ATRA did not affect cell proliferation or induce apoptosis of SW480 cells [61]. ATRA induced MUC-2 expression and alkaline phosphatase activity in SW480 cells, and AP 100984 reduced the effect [61].

### 3.3. Pancreatic Cancer and Hepatoma

A comprehensive study revealed CysLT_1_R expression in several pancreatic cancer cell lines (PA-TU-8988T, SUIT-2 and PANC1 cells, but not in MIAPaCa-2 cells); LTD_4_ promoted the proliferation of pancreatic cancer cells, whereas treatment with montelukast caused cell cycle arrest at G0/G1 phase without inducing apoptosis [62].

In a recent study using human hepatoma cell lines, CysLT antagonists (pranlukast and montelukast) inhibited ADAM9 activity and upregulated level of membrane-bound MHC class I-related chain A (mMICA), which might facilitate natural killer cell-mediated cytotoxicity, suggesting the potential of using leukotriene receptor antagonists along with regorafenib in the treatment of hepatoma [63].

### 3.4. Urological Malignancies

Several studies have demonstrated CysLT_1_R expression in human cancer cells of renal cell carcinoma, bladder cancer, prostate cancer and testicular cancer [64,65,66]. Treatment with montelukast downregulated CysLT_1_R expression, reduced cell viability and induced early apoptosis in human prostate cancer cell lines (LNCaP, PC3, DU-145) [64,67], a human renal cell carcinoma cell line (Caki-1) [64,68], a human bladder cancer cell line (T24) [64,65] and a testicular cancer cell line (NEC-8) [64,66]. The effects were not observed in normal stromal prostate cell lines [67], normal proximal tubular endothelial cells (PRTEC) [68]. Montelukast also inhibited hypoxia-induced HIF-1α activation in prostate cancer cells, but the effect was not shown by pranlukast and zafirlukast, suggesting this effect was not mediated by the CysLT_1_R pathway [69]. MK591, a 5-LO inhibitor developed to inhibit leukotriene biosynthesis, induced apoptosis in LNCaP cells [70]. Inhibition of 5-LO by MK886 completely blocked 5-HETE production, inducing massive apoptosis in both hormone-responsive (LNCaP) and hormone-nonresponsive (PC3) human prostate cancer cells [71].

### 3.5. Breast Cancer

LTB_4_ and LTD_4_ inhibited MCF-7 breast cancer cell growth, and a leukotriene antagonist (LY171883) and a 5-LO inhibitor (MK886) further lifted the inhibitory effect, suggesting that LT-receptors mediated the growth-inhibitory effect of LTB_4_ or LTD_4_ [72]. CysLTR antagonists, montelukast and zafirlukast reduced cell viability of MDAMB-231, a triple-negative breast cancer cell line, in a dose-dependent manner; zafirlukast mainly exerted its action on cell cycle, while montelukast mainly induced apoptosis [73].

Similar to the findings in colon cancer, activating CysLT_2_R signaling with LTC_4_ (preferentially binds to CysLT_2_R, rather than CysLT_1_R) did not affect cell proliferation or apoptosis of breast cancer cells but reduced cell migration [74].

### 3.6. Lung Cancer

Inhibition of 5-LO resulted in interruption of 5-LO-mediated growth factor signaling, resulting in significant growth reduction and enhanced apoptosis in a number of lung cancer cell lines [75]. Furthermore, 5-LO inhibitors (AA861 or ETH 615-139) and zafirlukast (a CysLT_1_R antagonist) blocked the release of organic osmolytes (taurine, meAIB) and the concomitant cell volume restoration following hypoosmotic swelling of A549 cells; inhibition of 5-LO or CysLT_1_R did not affect caspase-3 activity during hypoxia [76].

Montelukast inhibited the viability/proliferation of several lung cancer cell lines (A549, H460, H1299, CL1-0 and CL1-5), and induced cell death via nuclear translocation of apoptosis-inducing factor (AIF) [77].

### 3.7. Neurological Malignancies

A study examined several neuroblastoma cell lines (SH-SY5Y, SK-N-BE2, SK-N-SH, SK-N-AS, SK-N-FI, SK-N-DZ, IMR-32) and found that all of them expressed 5-LO, CysLT_1_R and CysLT_2_R [78]. Neuroblastoma cells endogenously produced leukotrienes [78]. LTD_4_ significantly increased cell viability and proliferation of neuroblastoma cells, and montelukast induced cell cycle arrest and apoptosis [78].

Montelukast and zafirlukast inhibited proliferation and induced apoptosis of glioblastoma cells (A172 and U-87 MG cells) in a concentration-dependent manner; both medications decreased Bcl-2 expression without affecting Bax level [79]. Montelukast induced more apoptosis than zafirlukast in A172 cells, but not in U-87 MG cells; zafirlukast caused cell cycle arrest at G0/G1 phase by upregulating the expression of p53 and p21 and showed a greater antiproliferative effect than montelukast [79]. Montelukast and zafirlukast, but not zileuton, significantly inhibited migration and invasion of glioblastoma cells, as well as inhibiting the expression and activities of MMP-2 and MMP-9 in glioblastoma cells and primary human astrocytes, suggesting the important role of CysLT_1_R in migration and invasion of glioblastoma [80].

### 3.8. Other Malignancies

Uveal melanoma cell lines derived from primary (Mel285, Mel270) and metastatic (OMM2.5) uveal melanoma expressed CysLT_1_R and CysLT_2_R [81]. Montelukast, quininib and 1,4-dihydroxy quininib significantly inhibited uveal melanoma cells in a time- and dose-dependent manner, whereas a CysLT_2_-selective antagonist, HAMI 3379, did not show growth inhibition effect [81]. Quininib significantly inhibited long-term proliferation, altered the cancer secretome of inflammatory and angiogenic factors and inhibited oxidative phosphorylation [81].

LTC_4_ mediated the second wave of Rac1 activation and cell migration; treatment with 5-LO inhibitors (AA861 and BU-4664L) or CysLT_1_R antagonists (MK571 and montelukast), as well as knockdown of CysLT_1_R, suppressed cell migration of A431 cells, an epidermoid carcinoma cell line, through downregulating EGF-induced expression of T cell lymphoma invasion and metastasis-inducing protein 1 (Tiam1) [82].

### 3.9. Drug Resistance and Cysteinyl Leukotriene Pathway

The LTD_4_ receptor has been highly associated with multidrug resistance protein (MRP), a membrane transporter of multiple anticancer drugs. MK571, a CysLT_1_R antagonist, modulated MRP-associated multidrug resistance in HL60/AR and GLC4/ADR cells, MRP-overexpressing multidrug resistant human leukemia and human small cell lung cancer cell lines, respectively, in a dose-dependent manner [83]. Similarly, increased expression of multidrug-resistance-associated protein 1 (MRP1) was observed in multidrug resistant phenotype of prostate cancer, and adding non-toxic doses of MK571, zafirlukast or buthionine sulfoximine significantly increased the sensitivity of the MDR models to cytotoxic drugs [84].

ONO-1078, a peptide LTD_4_ receptor antagonist, inhibited the transporting activity of MRP and increased vincristine uptake, resulting in increased sensitivity to vincristine of multidrug-resistant CV60 and its parental drug-sensitive KB-3-1 cell line [85]. Similarly, ONO-1078 enhanced the sensitivity of lung cancer NCI-H520 cells to several chemotherapeutic agents, including vincristine, doxorubicin and etoposide, through inhibiting the function of MRP [86].

To investigate the role of CysLT_1_R in chemoresistance, a study established 5-FU-resistant colon cancer cell lines by culturing with increasing concentration of 5-FU over a period of 6–8 months [87]. The 5-FU-resistant colon cancer cell lines expressed increased CysLT_1_R, which regulated 5-FU resistance via β-catenin activation and promoted 5-FU-resistance-derived stemness [87]. Montelukast restricted the motility of 5-FU-resistant colon cancer cells, sensitized them to 5-FU and decreased 5-FU-resistance-derived stemness, suggesting that inhibition of CysLT_1_R signaling with montelukast might reverse drug resistance of colon cancer [87].

## 4. Animal Studies about the Roles of Cysteinyl Leukotriene Pathway in Cancer

Major findings of animal studies about the roles of the CysLT pathway in cancer are summarized in Table A2 in Appendix A.

### 4.1. The Role of Vascular Permeability Mediated by Cysteinyl Leukotrienes

CysLTs may modulate vascular permeability, and therefore may affect drug delivery or tumor metastasis. An animal study using a rat C6 glioma model showed that LTE_4_ selectively opened the blood-tumor barrier and increased the tumor uptake of intravenously injected methotrexate [88].

In contrast, CysLTR antagonists may inhibit tumor metastasis by inhibiting capillary permeability. An animal study using transplantable rat colon adenocarcinoma RCN9 cells implanted via the cisterna magna of male Fisher rats showed that pranlukast, but not montelukast, could inhibit tumor cell migration through brain capillary [89]. Using Lewis lung carcinoma metastasis model in mice, the effect of both pranlukast and montelukast on inhibiting tumor cell migration through peripheral capillary was demonstrated [89].

A study using male C57BL/6 mice, including wild type, *Cysltr1*^−/−^ and *Cysltr2*^−/−^, implanted with Matrigel plugs or subcutaneously injected with Lewis lung carcinoma cells demonstrated the important role of CysLT_2_R in regulating tumor angiogenesis, metastasis and endothelial cell dysregulation [90]. CysLT_2_R-regulated angiogenesis was shown in isolated mouse endothelial cells and in Matrigel implants [90]. The growth and metastases of implanted Lewis lung carcinoma cells were significantly reduced in *CysLT_2_R*-null mice, compared with wild-type or *CysLT_1_R*-null mice. In wild-type mice subcutaneously implanted with Lewis lung carcinoma cells, the expression of CysLT_2_R, but not CysLT_1_R, was increased in tumor vasculature, while BayCysLT_2_ (a selective CysLT_2_R antagonist), but not MK571 (a CysLT_1_R antagonist), reduced tumor growth, angiogenesis and lung metastasis of tumor cells [90].

### 4.2. Colorectal Cancer

The role of CysLTs in colon cancer have been investigated in several studies using a xenograft model of nude mouse subcutaneously injected with human colon cancer cells. LTD_4_ promoted cancer-initiating cells in initiating tumor growth by allowing immuno-modulation of the tumor microenvironment [91]. The important role of CysLT_1_R in colon tumorigenesis was shown in a study using a colitis-associated colon cancer mice model induced by azoxymethane/dextran sulfate sodium, showing higher relative body weight, reduction in inflammation and polyps with lower-grade dysplasia and decreased nuclear expression of β-catenin and COX-2 in mice with global disruption of *CYSLTR1* gene expression [92]. In studies using a xenograft model, CysLT_1_R antagonists (montelukast and ZM198,615) inhibited proliferation and induced apoptosis of tumor cells, resulting in reduced size of the inoculated tumor [57,93]. Montelukast also significantly decreased amounts of cancer-stem cells, as well as macrophage infiltration and decreased expression of *ALDH1A1*, *DCLK1* and *BCL2* in the tumor tissue [57]. As CysLTs might involve in angiogenesis, 1,4-dihydroxy quininib significantly reduced tumor volume and the expression of angiogenic marker calpain-2 [58].

### 4.3. Pancreatic Cancer

The chemo-preventive effect and therapeutic potential of montelukast on pancreatic cancer was studied with a Syrian golden hamster model, using N-nitrosobis (2-oxopropyl) amine (BOP) to induce pancreatic ductal carcinomas, which were shown to be similar to those in humans morphologically and molecularly; montelukast suppressed pancreatic carcinogenesis by suppressing cell proliferation via the LTD_4_-CysLT_1_R axis [62].

### 4.4. Lung Cancer

The effects of leukotriene pathway inhibitors (zafirlukast, MK886 and Zileuton) on preventing lung cancer were investigated with a mice model of lung tumors induced by vinyl carbamate injection; leukotriene pathway inhibitors (zafirlukast, MK886 and zileuton) prevented lung tumor formation and slowed the growth and progression of adenomas to carcinoma [94]. A later study, using an orthotopic immunocompetent mouse model of lung cancer with Lewis lung carcinoma cells injected into lungs of syngeneic mice, found increased production of leukotrienes (LTB_4_, LTC_4_, LTD_4_ and LTE_4_), in dependent on cytosolic phospholipase A_2_, in the tumor microenvironment [95]. Our study using mice with Lewis lung carcinoma cells injected subcutaneously (to monitor the tumor growth on a daily basis) showed that montelukast significantly hampered the tumor growth, and the tumor tissue of the montelukast group showed markedly decreased Ki-67 expression and markedly increased terminal deoxynucleotidyl transferase dUTP nick end labeling (TUNEL)-positive cells [77].

### 4.5. Neurological Malignancies

An animal study using experimental brain tumor of intracerebral C6 glioma in a Sprague-Dawley rat model found that pretreatment with LTC_4_ significantly extends survival in rats treated with cisplatin [96].

## 5. Clinical Studies about the Roles of Cysteinyl Leukotriene Pathway in Cancer

Major findings of clinical studies about the roles of the CysLT pathway in cancer are summarized in Table A3 in Appendix A.

### 5.1. Hematological Malignancies

A study analyzing blood samples from 17 CML patients in the chronic phase and 15 healthy medication-free volunteers found that mature CML CD16 (+) neutrophils had aberrantly increased expression of LTC_4_ synthase, which might be responsible for stimulating the proliferation of human bone marrow-derived myeloid progenitor cells [97]. A subsequent study showed that urinary excretion of LTE_4_ was significantly higher in CML patients than healthy controls, supporting the increased LTC_4_ synthase activity in CML patients; they further found that neutrophilic LTC_4_ synthase expression and activity were markedly elevated and were normalized with imatinib mesylate treatment [98]. A study using multiplex single cell polymerase chain reaction analyzed the expression of the mediators of the leukotriene pathway in bone marrow BCR-ABL^+^CD34^+^CD38^−^ cells at diagnosis found the majority of cells expressed CysLT_1_R and CysLT_2_R, but not ALOX5; treatment with zileuton or montelukast failed to suppress cell growth, suggesting that targeting the CysLT pathway may not be a promising strategy to eradicate leukemia stem cells in CML patients [99].

The lymph node biopsy specimens from 57 non-Hodgkin lymphoma patients were assessed using immunohistochemical staining, and it was found that primary mediastinal B-cell lymphoma was the only type showing CysLT_1_R expression in tumor cells, while other types of lymphoma included in the study showed no CysLT_1_R expression [42]. The same study group further assessed biopsy specimens from 29 Hodgkin lymphoma patients with immunohistochemical studies and assessed the micro-dissected Hodgkin Reed–Sternberg cells with microarray analysis, showing that CysLT_1_R was expressed by primary Hodgkin Reed–Sternberg cells, which were surrounded by CysLT-producing eosinophils, macrophages and mast cells; the findings might suggest that CysLTs were important mediators in the pathogenesis of Hodgkin lymphoma, contributing to the aberrant cytokine network [43].

### 5.2. Colorectal Cancer

Several studies have analyzed tissue samples from colorectal cancer patients with immunohistochemical staining, in situ hybridization and tissue microarrays, showing higher CysLT_1_R and 5-LO expression, particular in the nuclei, in the cancer tissues than in normal colon tissues [54]. Nuclear accumulation of CysLT_1_R was strongly correlated with stronger staining of proliferative marker Ki-67 [54]. Higher expression of CysLT_1_R, particularly in the nucleus, was associated with a poorer prognosis [44,100], whereas higher nuclear expression of CysLT_2_R was associated with an earlier stage and better prognosis [59,100]. Aggressive tumors generally expressed less CysLT_2_R and IFN-α receptor and more EGFR, with a negative correlation between CysLT_2_R and EGFR expression, suggesting a potential protective role of CysLT_2_R against tumor progression [60]. In addition, 1,4-dihydroxy quininib significantly decreased the secretion of both angiogenic factor TIE-2 and adhesion molecule VCAM-1 in human ex vivo colorectal cancer tumor explants [58].

### 5.3. Esophageal Cancer and Gastric Cancer

A study assessed esophageal biopsy specimens with specific enzyme immunoassays found higher expression levels of LTB_4_ and CysLTs in esophageal adenocarcinoma tissue compared to Barrett’s metaplasia and control tissues [101]. However, another study analyzing esophageal biopsy samples with immunohistochemistry and quantitative reverse transcription-polymerase chain reaction showed significantly lower expression levels of CysLT_1_R and CysLT_2_R in esophageal cancer tissues than in control squamous epithelium [102].

Assessing with immunohistochemistry, gastric cancer tissue showed significantly increased immunoreactive score of CysLT_1_R than tumor-free gastric mucosa; the intestinal type had more CysLT_1_R and CysLT_2_R expression than the diffuse type [103].

### 5.4. Pancreatic Cancer and Hepatoma

In a study enrolling 92 hepatocellular carcinoma patients and 20 healthy control subjects, remarkably higher circulating LTD_4_ level was noted in patients of hepatocellular carcinoma, particular in those with chronic hepatitis B infection or metastasis [104].

Immunohistochemical analyses of 108 pancreatic ductal adenocarcinoma tissues revealed that high CysLT_1_R expression was associated with worse overall survival [62].

### 5.5. Urological Malignancies

Strong CysLT_1_R expression was noted in several urological cancer samples, including renal cell carcinoma [64,68], bladder cancer (transitional cell carcinoma) [64,65], prostate cancer and prostatic intraepithelial neoplasia [64,67] and testicular cancer (including seminoma, embryonal carcinoma, yolk sac tumors, choriocarcinoma and teratoma) [64,66], than in the corresponding normal tissue, and more extensive and intense expression was noted in cancer with a higher grade or an advanced stage. Very weak CysLT_1_R expression was observed in relatively normal tissues (normal kidney tissue, normal bladder tissue, benign prostatic hyperplasia, normal prostate tissue and normal testis tissue) [64].

### 5.6. Breast Cancer

A study investigating 144 breast cancer specimens with tissue microarray and immunohistochemistry found that breast cancers with higher CysLT_1_R and lower CysLT_2_R expression levels were associated with higher histological grade and worse overall survival [74].

### 5.7. Other Malignancies

CysLT_1_R staining was evident in neuroblastoma surgical specimens and the adjacent vasculature, suggesting a potential treatment target [78].

A study assessing data of 80 primary uveal melanoma samples in The Cancer Genome Atlas (TCGA) showed that higher expression of *CYSLTR1* and *CYSLTR2* genes were significantly associated with poorer disease-free survival and overall survival [81]. However, the study group also examined the tissue from 52 patients of primary uveal melanoma using tissue microarray and only found poorer overall survival in those with higher CysLT_1_R expression, whereas CysLT_2_R expression was not associated with survival [81].

### 5.8. Chemopreventive Effects of Cysteinyl Leukotriene Inhibition

A large nationwide retrospective cohort study using the Taiwan National Health Insurance Research Database investigated the effect of CysLTR antagonists (LTRAs), including montelukast and zafirlukast, in reducing cancer incidence in asthma patients [105]. Comparing 4185 LTRA users with 20,925 LTRA non-users, we found that using LTRA decreased cancer risk in a dose-dependent manner, and the chemo-preventive effect of LTRA was markedly observed in terms of lung, colorectal, liver and breast cancer [105]. Another national retrospective cohort study used data from the Department of Veteran Affairs to investigate the association between leukotriene inhibition and lung cancer incidence among U.S. Veterans with asthma [106]. The researchers found that 23,730 patients with leukotriene pathway inhibiting medication (montelukast, zafirlukast, or zileuton) exposure, compared with 534,736 patients without exposure, had significantly reduced risk of lung cancer [106].

## 6. Future Perspectives

In summary, inflammation plays important roles in tumor initiation, progression and metastasis [6], and the role of the CysLT pathway in cancer has been demonstrated in several cancers using different approaches. Increased 5-LO expression and downstream CysLT pathway have been recognized as inflammation to shape the tumor microenvironment and are associated with cancer proliferation, migration and invasion. 

Serial change in the expression level of CysLT_1_R within the spectrum from normal tissue to low-grade and then advanced malignancies has been documented in several cancers. For example, very weak CysLT_1_R expression was observed in relatively normal tissues or benign lesions of urological organs [64]. In contrast, strong CysLT_1_R expression was noted in several urological cancer samples, with higher expression associated with a higher tumor grade or an advanced stage [64,65,66,67,68]. Similarly, higher CysLT_1_R and 5-LO expression was noted in colon cancer tissues than in normal colon tissues, with higher expression level of CysLT_1_R associated with higher tumor Ki-67 level and poorer prognosis [44,54,100]. The role of CysLT_1_R in colon tumorigenesis was demonstrated in a study using a colitis-associated colon cancer mice model, showing polyps with lower-grade dysplasia in mice with disruption of *CYSLTR1* expression [92]. These findings demonstrated the important role of the CysLT pathway in carcinogenesis and tumor progression.

While the findings still appear somewhat heterogenous, further studies are warranted to clarify the role of the CysLT pathway in cancer biology, including cancer cells and their microenvironment, of different cancers. In our opinion, the following aspects require further investigation: (1) the roles of CysLTs and CysLTRs in proliferation, stemness, invasion and migration of cancer cells, as well as their secretomes and interactions with other cells in the tumor microenvironment; (2) the roles of CysLTs and CysLTRs in the cells in tumor microenvironment, particularly tumor-associated immune cells, which establish a cancer-promoting milieu; (3) the effects of CysLT inhibition, particularly CysLTR antagonists, in chemoprevention; and (4) the effects of CysLT inhibition, particularly CysLTR antagonists, as adjunct for the currently available standard anti-cancer treatment modalities. With more understanding about the role of the CysLT pathway in cancer, we will have better chance to provide precision care, including diagnostic and therapeutic approaches, for cancer patients.

## Figures and Tables

**Table 1:** Cysteinyl leukotriene pathway antagonists.

Medication	Pharmacologic Effect	Remark
Zileuton	5-LO inhibitor	Clinically available *
AA861	5-LO inhibitor	
BU-4664L	5-LO inhibitor	
BWA4C	5-LO inhibitor	
ETH 615-139	5-LO inhibitor	
MK591	5-LO inhibitor	
MK886	5-LO inhibitor	
Montelukast	Selective CysLT_1_R antagonist	Clinically available *
Zafirlukast	Selective CysLT_1_R antagonist	Clinically available *
Pranlukast	Selective CysLT_1_R antagonist	Clinically available *
MK571	Selective CysLT_1_R antagonist	
ZM198,615	Selective CysLT_1_R antagonist	
AP 100984	Selective CysLT_2_R antagonist	
BayCysLT_2_	Selective CysLT_2_R antagonist	
HAMI 3379	Selective CysLT_2_R antagonist	
Quininib 1,4-dihydroxy quininib	Selective CysLT_1_R (and CysLT_2_R) antagonist	

Abbreviations: 5LO = 5-lipoxygenase; CysLT_1_R = cysteinyl leukotriene 1 receptor; CysLT_2_R = cysteinyl leukotriene 2 receptor. * Approved by the US Food and Drug Administration.

## Data Availability

Not applicable.

## Appendix A

**Table A1:** Selected in vitro studies investigating the cysteinyl leukotriene pathway in cancer.

Author, Year	Cancer Cells	Major Findings
Hematologic malignancies		
Stenke L, 1988 [37]	chronic myelogenous leukemia cell	Increased LTC_4_ synthase activity with increased LTC_4_ production in chronic myelogenous leukemia cells.
Stenke L, 1990 [38]	chronic myelogenous leukemia cell	Increased LTC_4_, rather than LTB_4_, producing capacity in patients of chronic myelogenous leukemia.
Chan CC, 1994 [16]	THP-1, a human monocytic leukemia cell line	LTD_4_ induced increased cytosolic calcium in THP-1 cells.
Hoshino M, 1998 [17]	THP-1, a human monocytic leukemia cell line	LTD_4_ activated the MAPK pathway.
Yektaei-Karin E, 2017 [39]	chronic myeloid leukemia (CML) cell lines (K562, KCL22, KU812), primary CD34 + blood cells from two CML patients, human colon carcinoma cell lung (HCT-116), lung fibroblast cell line (WI-38)	Montelukast and BWA4C (a 5-LO inhibitor) reduced cell proliferation of CML cells in a dose dependent manner.
Zovko A, 2018 [40]	chronic myeloid leukemia cell lines (K562 and JURL-MK1)	Montelukast, through a CysLT_1_R-dependent pathway, induced apoptosis of CML cells by inducing Bax overexpression, cytochrome c release, PARP-1 cleavage, and caspase-3 activation, as well as altered Wnt/β-catenin signaling.
Drost AC, 2012 [41]	chronic lymphocytic leukemia cell lines (EHEB, MEC-1); primary chronic lymphocytic leukemia cells from 54 patients; CD19 + (B cells) and CD19-cells from 8 healthy individuals	EHEB and MEC-1 cells expressed high levels of CysLT_1_R and low level of CysLT_2_R. LTD_4_ induced CysLT_1_R-mediated calcium fluxes, actin polymerization, chemotaxis and activation of MAPK pathway in chronic lymphocytic leukemia (CLL) cells. CysLT_1_R antagonists (MK571 and LY171883) reduced viability and increased apoptosis of CLL cells.
Schain F, 2008 [42]	human primary mediastinal B-cell lymphoma cell lines (Med-B1, Karpas-1106P) and a Hodgkin lymphoma cell line (L1236)	Med-B1, Karpas-1106P and L1236 cells expressed CysLT_1_R. LTC_4_ and LTD_4_ induced a calcium signal in Med-B1 cells, which could be blocked by zafirlukast.
Schain F, 2008 [43]	human Hodgkin lymphoma cell line cell lines (L1236, HDLM2, KMH2, L428, L591)	L1236 and KMH2 cells expressed functional CysLT_1_R, responding with a robust calcium signal upon LTD_4_ challenge, which could be blocked by zafirlukast.
Colorectal cancer		
Ohd JF, 2003 [44]	Int 407 and two human colon cancer cell lines (Caco-2 and SW-480)	Int 407, Caco-2 and SW-480 expressed CysLT_1_R. The viability of Caco-2 cells increased with overexpression of CysLT_1_R.
Nielsen CK, 2003 [45]	Intestine 407 (Int 407), a human embryonic intestinal epithelial cell line	LTD_4_ prevented apoptosis of Int 407 cells induced by NS-398, a COX-2 inhibitor.
Wikström K, 2003 [46]	Intestine 407 (Int 407), a human embryonic intestinal epithelial cell line	LTD_4_ prevented apoptosis of Int 407 cells induced by NS-398, a COX-2 inhibitor. LTD_4_ also reduced the apoptotic potential by preventing caspase 8 activation and Bid cleavage.
Paruchuri S, 2002 [47]	Intestine 407 (Int 407), a human embryonic intestinal epithelial cell line	LTD_4_ enhance proliferation of Int 407 cells via two distinct signaling pathways (a Ras-independent and a Ras-dependent).
Wikström K, 2003 [48]	a human embryonic intestinal epithelial cell line (Int 407) and a human colorectal carcinoma cell line (Caco-2)	LTD_4_ upregulated COX-2 and Bcl-2 through a pertussis toxin sensitive G-protein and MAPK pathway in Int 407 and Caco-2 cells.
Parhamifar L, 2005 [49]	a human embryonic intestinal epithelial cell line (Int 407) and a human colorectal carcinoma cell line (Caco-2)	LTD_4_ stimulation induced cytosolic phospholipase A2α activation and nuclear translocation via a calcium-dependent mechanism involving activation of PKC and the MAPK pathway in both Int 407 and Caco-2 cells.
Mezhybovska M, 2006 [50]	Intestine 407 (Int 407), a human embryonic intestinal epithelial cell line	LTD_4_ induced β-catenin signaling in Int 407 cells, which activated TCF/LEF transcription factors in the nucleus and increased the association between β-catenin with Bcl-2 in the mitochondria.
Mezhybovska M, 2009 [51]	a human embryonic intestinal epithelial cell line (Int 407) and a human colorectal carcinoma cell line (Caco-2)	LTD_4_ increased mitochondrial metabolic activity and gene transcription and increased reactive oxygen species levels and subsequent activations of the p65 subunit of NF-κB, presumably through β-catenin accumulation in the mitochondria.
Paruchuri S, 2005 [52]	Intestine 407 (Int 407), a human embryonic intestinal epithelial cell line	LTD_4_ trigger a motile response of Int 407 cells via a PI3K/Rac signaling pathway.
Salim T, 2014 [53]	Human colorectal adenocarcinoma cell lines (HCT116, HT29)	LTD_4_ increased β-catenin level in colon cancer cells. In HCT116 cells but not in HT29 cells, LTD_4_ induced nuclear translocation of β-catenin, upregulated β-catenin target genes, and enhanced proliferation and migration; the effect could be prevented by pretreatment with ZM198,615, a CysLT_1_R antagonist.
Nielsen CK, 2005 [54]	a human embryonic intestinal epithelial cell line (Int 407) and a human colorectal carcinoma cell line (Caco-2)	CysLT_1_Rs were found in the outer nuclear membrane in Int 407 and Ca-co-2 cells. LTD_4_ induced nuclear translocation of the CysLT_1_Rs from the plasma membrane to the nucleus in Int 407 cells.
Yudina Y, 2008 [55]	Int 407 and 4 colon cancer cell lines (HT-29, SW-480, Caco-2, HCT-116)	The basal level of CysLT_1_R was higher in colon cancer cells compared to Int 407 cells. LTD_4_ significantly increased CysLT_1_R expression in Int 407 cells, but not in colon cancer cell lines; LTD_4_ induced upregulation of CysLT_2_R in colon cancer cell lines.
Paruchuri S, 2006 [56]	non-tumor intestinal epithelial cell lines (Int 407 and IEC-6) and tumor intestinal cell lines (Caco-2, SW480, HCT-116 and HT-29)	Constitutive CysLT_1_R signaling, which was maintained in an autocrine pattern, mediated both survival and proliferation of intestinal cells. A CysLT_1_R antagonist (MK571) induced apoptosis in non-tumor intestinal cells, but not in tumor-derived intestinal cell lines.
Bellamkonda K, 2018 [57]	human colon adenocarcinoma-derived cell lines (HT-29, SW-480)	Montelukast prevented LTD_4_-induced colony formation and disrupted colonospheres, as well as downregulation of cancer stem cell markers (ALDH1 and DCLK1).
Magnusson C, 2007 [59]	Int 407 and two human colon cancer cell lines (Caco-2 and SW-480)	The expression of CysLT_2_R, compared with CysLT_1_R, was higher in Int 407 but lower in two colon cancer cell lines (Caco-2 and SW480). CysLT_2_R was found to be located both at the plasma membrane and the nuclear membrane. CysLT_2_R signaling led to terminal differentiation of Caco-2 cells but had no effect on cell proliferation or apoptosis.
Magnusson C, 2011 [60]	Int 407 and 2 colon cancer cell lines (Caco-2, SW-480)	IFN-α could upregulate CysLT_2_R in Caco-2 cells. LTC_4_ induced expression mucin-2, and the effect could be blocked by AP 100984 (a specific CysLT_2_R antagonist) but not by montelukast (a specific CysLT_1_R antagonist). CysLT_2_R signaling was able to suppress cell migration induced by EGF signaling in Int 407 cells.
Bengtsson AM, 2013 [61]	colon cancer cell lines (Caco-2, SW-480, HCT-116)	All-trans retinoic acid (ATRA) treatment increased CysLT_2_R expression without affecting CysLT_1_R level, and upregulated LTC_4_ synthase in SW480 cells; the effect was not observed in HCT-116 cells. ATRA did not affect cell proliferation or induce apoptosis of SW480 cells. ATRA induced MUC-2 expression and alkaline phosphatase activity in SW480 cells, and a CysLT_2_R antagonist (AP 100984) reduced the effect.
Butler CT, 2019 [58]	HT29-Luc2 Bioware Ultra human colorectal cancer cell line	1,4-dihydroxy quininib reduced clonal formation of HT29-Luc2. Gene silencing of CysLT_1_R in HT29-Luc2 cells significantly reduced expression of angiogenic marker calpain-2.
Pancreatic cancer and hepatoma		
Kachi K, 2021 [62]	human pancreatic cancer cell lines (PA-TU-8988T, MIAPaCa-2, SUIT-2, PANC1)	CysLT_1_R was expressed in PA-TU-8988T, SUIT-2 and PANC1 cells, but not in MIAPaCa-2 cells. LTD_4_ promoted pancreatic cancer cell proliferation, whereas treatment with montelukast caused cell cycle arrest at G0/G1 phase without inducing apoptosis.
Arai J, 2021 [63]	human hepatoma cell lines (HepG2, PLC/PRF/5)	Pranlukast and montelukast inhibited ADAM9 activity and upregulated level of mem-brane-bound MHC class I-related chain A (mMICA).
Urological malignancies		
Matsuyama M, 2010 [64]	a human RCC cell line (Caki-1), normal prostate stromal cells, a human bladder cancer cell line (T24), human prostate cancer cell lines (LNCaP, PC3, DU-145), a testicular cancer cell line (NEC-8), normal proximal tubular endothelial cells	All tested cancer cell lines showed CysLT_1_R expression, and treatment with montelukast downregulated CysLT_1_R expression, reduced cell viability, and induced early apoptosis in these cells.
Matsuyama M, 2009 [65]	T24, a human transitional cell carcinoma cell line	T24 cells expressed CysLT_1_R; montelukast induced apoptosis of T24 cells.
Matsuyama M, 2009 [66]	NEC-8, a human testicular cancer cell line	NEC-8 expressed CysLT_1_R. Montelukast reduced cell viability and induced apoptosis of NEC-8 cells.
Matsuyama M, 2007 [67]	human prostate cancer cell lines (LNCaP, PC3, DU-145) and normal stromal prostate cell lines	Montelukast significantly inhibited the proliferation and induced apoptosis of prostate cancer cell lines, but not normal stromal prostate cell lines.
Funao K, 2008 [68]	human renal cell carcinoma cell line (Caki-1) and normal proximal tubular endothelial cells	Montelukast significantly inhibited the proliferation and induced apoptosis of Caki-1, but not normal proximal tubular endothelial cells.
Tang C, 2018 [69]	human prostate cancer cell lines (LNCaP, PC-3, DU-145) and normal stromal prostate cell lines (NPCs)	Montelukast inhibited hypoxia-induced HIF-1α activation in prostate cancer cells, and inhibited their proliferation. Montelukast also induced downregulation of HIF-1α under hypoxic conditions, but the effect was not shown by pranlukast and zafirlukast.
Sarveswaran S, 2010 [70]	LNCaP, a human prostate cancer cell line	MK591, a 5-LO inhibitor developed to inhibit leukotriene biosynthesis, induced apoptosis in LNCaP cells.
Ghosh J, 1998 [71]	Hormone-responsive (LNCaP) and -nonresponsive (PC3) human prostate cancer cells	Inhibition of 5-LO by MK886 induced massive apoptosis in human prostate cancer cells, LNCaP and PC3.
Breast cancer		
Przylipiak A, 1998 [72]	MCF-7, a human mammary cancer cell line	LTB_4_ and LTD_4_ inhibited growth of MCF-7 cells, and leukotriene antagonist LY171883 and 5-LO inhibitor MK886 further lifted the inhibitory effect.
Suknuntha K, 2018 [73]	MDAMB-231, a triple-negative breast cancer cell line	Montelukast and zafirlukast reduced cell viability of MDAMB-231 in a dose-dependent manner. Montelukast mainly induced apoptosis, while zafirlukast mainly exerted its action on cell cycle.
Magnusson C, 2011 [74]	breast cancer cell lines (MCF-7, MDA-MB-231)	Activating CysLT_2_R signaling with LTC_4_ did not affect cell proliferation or apoptosis of breast cancer cells but reduced cell migration.
Lung cancer		
Avis IM, 1996 [75]	human small cell lung cancer cell lines (H209, H345, H82, N417) and human non-small cell lung cancer cell lines (H1155, H23, A549)	Inhibition of 5-LO resulted in enhanced levels of programmed cell death and significant growth reduction for a number of lung cancer cell lines.
Holm JB, 2013 [76]	A549, a human adenocarcinoma cell line	5-LO inhibitors (AA861 or ETH 615-139) and zafirlukast (a CysLT_1_R antagonist) blocked the release of organic osmolytes (taurine, meAIB) and the concomitant cell volume restoration following hypoosmotic swelling of A549 cells; inhibition of 5-LO or CysLT_1_R did not affect caspase-3 activity during hypoxia.
Tsai MJ, 2017 [77]	human lung cancer cell lines (A549, H460, H1299, CL1-0, CL1-5) and mouse Lewis lung carcinoma cell line (LLC1)	Montelukast inhibited the viability/proliferation of lung cancer cells and induced cell death via nuclear translocation of apoptosis-inducing factor.
Neurological malignancies		
Sveinbjörnsson B, 2008 [78]	human neuroblastoma cell lines (SH-SY5Y, SK-N-BE2, SK-N-SH, SK-N-AS, SK-N-FI, SK-N-DZ, IMR-32) and a human myelocytic cell line (U937)	All neuroblastoma cell lines expressed CysLT_1_R and CysLT_2_R. Neuroblastoma cells endogenously produced leukotrienes. LTD_4_ significantly increased cell viability and proliferation of neuroblastoma cells. Montelukast induced cell cycle arrest and apoptosis of neuroblastoma cells.
Piromkraipak P, 2018 [79]	glioblastoma cell lines (A172, U-87 MG)	Montelukast and zafirlukast inhibited proliferation and induced apoptosis in a concentration-dependent manner in glioblastoma cells; both medications decreased Bcl-2 expression without affecting Bax level. Montelukast induced more apoptosis than zafirlukast in A172 cells, but not in U-87 MG cells; zafirlukast caused cell cycle arrest at G0/G1 phase by upregulating the expression of p53 and p21 and showed a greater antiproliferative effect than montelukast.
Piromkraipak P, 2018 [80]	glioblastoma cell lines (A172, U373) and primary human astrocyte	Montelukast and zafirlukast, but not zileuton, significantly inhibited migration and invasion of glioblastoma cells, as well as inhibited the expression and activities of MMP-2 and MMP-9.
Other malignancies		
Slater K, 2020 [81]	uveal melanoma cell lines derived from primary (Mel285, Mel270) and metastatic (OMM2.5) uveal melanoma	Uveal melanoma cell lines expressed CysLT_1_R and CysLT_2_R. Montelukast, quininib, and 1,4-dihydroxy quininib significantly inhibited uveal melanoma cells in a time- and dose- dependent manner, whereas a CysLT_2_-selective antagonist, HAMI 3379, did not show growth inhibition effect. Quininib significantly inhibited long-term proliferation, altered the cancer secretome of inflammatory and angiogenic factors, and inhibited oxidative phosphorylation.
Magi S, 2013 [82]	A431, an epidermoid carcinoma cell line	LTC_4_ mediated the second wave of Rac1 activation and cell migration; treatment with 5-LO inhibitors (AA861 and BU-4664L) or CysLT_1_R antagonists (MK571 and montelukast), as well as knockdown of CysLT_1_R, suppressed cell migration of A431 cells.
Association between cysteinyl leukotriene pathway and drug resistance		
Gekeler V, 1995 [83]	multidrug resistant human leukemia cell line (HL60/AR) and human small cell lung cancer cell line (GLC4/ADR)	MK571 modulated MRP-associated multidrug resistance in HL60/AR and GLC4/ADR cells.
Brussel JP, 2004 [84]	PC3 and DU-145, human prostate cancer cell lines	Prostate cancer cell with multidrug resistant phenotype had increased expression of multidrug-resistance-associated protein 1 (MRP1) and adding MK571, zafirlukast or buthionine sulfoximine significantly increased the sensitivity to cytotoxic drugs.
Nagayama S, 1998 [85]	human epidermoid carcinoma cell line (KB-3-1) and its multidrug resistant subclone (CV 60)	ONO-1078, a LTD_4_ receptor antagonist, inhibited the transporting activity of MRP and increased the sensitivity to vincristin of CV 60 and KB-3-1 cells.
Nakano R, 1998 [86]	NCI-H520, a human lung cancer cell line	ONO-1078 enhanced the sensitivity of lung cancer NCI-H520 cells to vincristine, doxorubicin and etoposide through inhibiting the function of MRP.
Satapathy SR, 2020 [87]	human colon cancer cell lines (HCT116, HT-29) and their 5-FU-resistant (5-FU-R) cell lines	The 5-FU-R colon cancer cell lines expressed increased CysLT_1_R, which regulated 5-FU resistance via β-catenin activation and promoted 5-FU-R-derived stemness. Montelukast restricted the motility of 5-FU-R colon cancer cells, sensitized them to 5-FU, and decreased 5-FU-R-derived stemness.

**Table A2:** Selected animal studies investigating the cysteinyl leukotriene pathway in cancer.

Author, Year	Animal Model of Cancer	Major Findings
The role of vascular permeability mediated by cysteinyl leukotrienes		
Chio CC, 1995 [88]	Rat C6 glioma model	LTE_4_ selectively opened the blood-tumor barrier and increased the tumor uptake of intravenously injected methotrexate.
Nozaki M, 2010 [89]	male Fisher rats, transplantable rat colon adenocarcinoma RCN9 cells implanted via the cisterna magna; Lewis lung carcinoma metastasis model in mice	Pranlukast, but not montelukast, inhibited tumor cell migration through brain capillary; both pranlukast and montelukast inhibited tumor cell migration through peripheral capillary.
Duah E, 2019 [90]	male C57BL/6 mice, including wild type, *Cysltr1*^−/−^ and *Cysltr*2^−/−^, implanted with Matrigel plugs or subcutaneously injected with Lewis lung carcinoma (LLC) cells	CysLT_2_R regulated angiogenesis in isolated mouse endothelial cells and in Matrigel implants in mice. The growth and metastases of implanted LLC cells were significantly reduced in *CysLT_2_R*-null mice than in wild-type or *CysLT_1_R*-null mice. In wild-type mice, the expression of CysLT_2_R, but not CysLT_1_R, was increased in tumor vasculature, and BayCysLT_2_ (a selective CysLT_2_R antagonist), but not MK571 (a CysLT_1_R antagonist), reduced tumor growth, angiogenesis and lung metastasis of Lewis lung carcinoma cells in wild-type mice.
Colorectal cancer		
Bellamkonda K, 2018 [57]	a xenograft model of nude mice subcutaneously injected with human colon adenocarcinoma-derived cell lines (HT-29, SW-480)	Montelukast significantly inhibited tumor growth and decreased amounts of cancer-stem cells.
Bellamkonda K, 2016 [91]	a nude mouse xenograft model with subcutaneous injection of human HCT-116 colon cancer cells	LTD_4_ promoted cancer-initiating cells in initiating tumor growth by allowing immuno-modulation of the tumor microenvironment.
Osman J, 2017 [92]	a colitis-associated colon cancer mice model induced by azoxymethane/dextran sulfate sodium	The mice with global disruption of *CYSLTR1* gene expression had higher relative body weight, reduction in inflammation, and polyps with lower-grade dysplasia and decreased nuclear expression of β-catenin and COX-2.
Savari S, 2013 [93]	a xenograft model of nude mice inoculated with HCT-116 colon cancer cells	CysLT_1_R antagonists (montelukast and ZM198,615) inhibited proliferation and induced apoptosis of tumor cells, resulting in reduced size of the inoculated tumor.
Butler CT, 2019 [58]	HT29-Luc2 cells in immunocompromised mice (Balb/C nu/nu)	In the tumor xenograft model, 1,4-dihydroxy quininib significantly reduced tumor volume and the expression of angiogenic marker calpain-2.
Pancreatic cancer		
Kachi K, 2021 [62]	a Syrian golden hamster model, using N-nitrosobis (2-oxopropyl) amine (BOP) to induce pancreatic ductal carcinomas	Montelukast suppressed pancreatic carcinogenesis by suppressing cell proliferation via the LTD_4_-CysLT_1_R axis.
Lung cancer		
Tsai MJ, 2017 [77]	Mice with Lewis lung carcinoma injected subcutaneously	Montelukast significantly delayed the tumor growth, as well as decreased Ki-67 expression and increased terminal deoxynucleotidyl transferase dUTP nick end labeling (TUNEL)-positive cells in the tumor tissue.
Gunning WT, 2002 [94]	vinyl carbamate injection to induce lung tumors in mice	Leukotriene pathway inhibitors (zafirlukast, MK886, and Zileuton) prevented lung tumor formation and slowed the growth and progression of adenomas to carcinoma.
Poczobutt JM, 2013 [95]	orthotopic immunocompetent mouse model of lung cancer with Lewis lung carcinoma cells injected into lungs of syngeneic mice	A later study, using an orthotopic immunocompetent mouse model of lung cancer with Lewis lung carcinoma cells injected into lungs of syngeneic mice, found increased production of leukotrienes (LTB_4_, LTC_4_, LTD_4_ and LTE_4_), in dependent on cytosolic phospholipase A_2_, in the tumor microenvironment.
Neurological malignancies		
Black P, 1998 [96]	intracerebral C6 glioma in Sprague-Dawley rats	Pretreatment with LTC_4_ did significantly extend survival in rats treated with cisplatin

**Table A3:** Selected clinical studies investigating the cysteinyl leukotriene pathway in cancer.

Author, Year	Patients/Specimens	Major Findings
Hematological malignancies		
Schain F, 2008 [42]	lymph node biopsy specimens from 57 non-Hodgkin lymphoma patients	Primary mediastinal B-cell lymphoma was the only type showing CysLT_1_R expression in tumor cells, while other included lymphomas showed no CysLT_1_R expression.
Schain F, 2008 [43]	Specimens from 29 Hodgkin lymphoma patients	CysLT_1_R was expressed by primary Hodgkin Reed–Sternberg cells.
Sjölinder M, 2000 [97]	17 patients in the chronic phase of Ph chromosome-positive (Ph+) chronic myeloid leukemia (CML) and 15 healthy medication-free volunteers	Mature CML CD16 (+) neutrophils had aberrantly increased expression of LTC_4_ synthase.
Roos C, 2008 [98]	23 chronic myeloid leukemia (CML) patients and 18 healthy controls	Urinary excretion of LTE_4_ was significantly higher in CML patients than healthy controls. Neutrophilic LTC_4_ synthase expression and activity were markedly elevated and were normalized with imatinib mesylate treatment.
Dolinska M, 2017 [99]	bone marrow aspirates collected from 10 chronic myeloid leukemia (CML) patients and healthy adult volunteers	The majority of the single leukemic BCR-ABL^+^CD34^+^CD38^−^ cells expressed CysLT_1_R and CysLT_2_R, but not ALOX5; treatment with zileuton or montelukast failed to suppress cell growth.
Colorectal cancer		
Ohd JF, 2003 [44]	colorectal cancer samples from 84 patients	Using immunohistochemistry and in situ hybridization performed on tissue arrays showed that cysLT_1_R expression was significantly associated with poorer survival.
Nielsen CK, 2005 [54]	specimens from 44 patients with colorectal cancer	Colorectal cancer tissue showed higher CysLT_1_R and 5-LO immunohistochemical staining, particular in the nuclei, compared to normal colon tissue; the nuclear accumulation of CysLT_1_R was strongly correlated with stronger staining of proliferative marker Ki-67.
Magnusson C, 2007 [59]	tissues from 77 patients of colon cancer	Colon cancer tissue had a significantly lower CysLT_2_R expression than paired normal tissue; lower expression of CysLT_2_R in colon cancer was associated with later stage and poorer prognosis.
Magnusson C, 2011 [60]	specimens from 78 colon cancer patients	The tissue microarray showed that aggressive tumors generally expressed less IFN-α receptor and more EGFR, with a negative correlation between CysLT_2_R and EGFR expression.
Magnusson C, 2010 [100]	tissue microarray of 329 colorectal cancer patients	Higher nuclear expression of CysLT_1_R was associated with a poorer prognosis, whereas higher nuclear expression of CysLT_2_R was associated with a better prognosis.
Butler CT, 2019 [58]	human ex vivo colorectal tumor explants representing Dukes’ stage A, B and C colorectal cancer	In human ex vivo colorectal cancer tumor explants, 1,4-dihydroxy quininib significantly decreased the secretion of both angiogenic factor TIE-2 and adhesion molecule VCAM-1.
Esophageal cancer and gastric cancer		
Shutt JD, 2012 [101]	esophageal biopsy specimens from 14 Barrett’s metaplasia, 2 high-grade dysplasia, 11 esophageal adenocarcinoma and 11 squamous control	Increased expression levels of LTB_4_ and CysLTs were found in esophageal adenocarcinoma tissue than in Barrett’s metaplasia and control tissues.
Venerito M, 2016 [102]	19 patients with esophageal squamous cell cancer and 9 sex- and age-matched patients with functional dyspepsia	Significantly decreased expression levels of CysLT_1_R and CysLT_2_R were noted in esophageal cancer tissues compared with control squamous epithelium.
Venerito M, 2011 [103]	35 gastric cancer tissue, 29 tumor-free tissue	Gastric cancer tissue showed significantly increased immunoreactive score of CysLT_1_R than tumor-free gastric mucosa; intestinal type had more CysLT_1_R and CysLT_2_R expression than the diffuse type.
Zhou Y, 2011 [104]	92 hepatocellular carcinoma (HCC) patients and 20 health controls	Remarkably higher circulating LTD_4_ level was noted in HCC patients versus healthy subjects, especially in patients with chronic hepatitis B infection or metastasis.
Pancreatic cancer and hepatoma		
Kachi K, 2021 [62]	108 samples of pancreatic ductal adenocarcinoma tissues	Immunohistochemical analyses on pancreatic ductal adenocarcinoma tissues revealed that high CysLT_1_R expression was associated with worse overall survival.
Urological malignancies		
Matsuyama M, 2010 [64]	tissue specimens from: 58 patients with renal cell carcinoma, paired with normal kidney tissue from 20 patients; 90 patients with bladder cancer, paired with normal bladder tissue from 30 patients; 151 patients with prostate cancer, 20 patients with prostatic intraepithelial neoplasia, 20 patients with benign prostatic hyperplasia, paired with normal prostate tissue from 20 patients; 30 patients with testicular cancer, paired with normal testis tissue from 10 patients	Immunohistochemistry revealed strong CysLT_1_R expression in all cancer samples, and more extensive and intense expression was noted in cancer with a higher grade or an advanced stage. Very weak CysLT_1_R expression was observed in relatively normal tissues.
Matsuyama M, 2009 [65]	tissue specimens from 90 transitional cell carcinoma (TCC) patients and 30 patients with normal bladder	CysLT_1_R expression was significantly more extensive and intense in TCC specimens, but not in normal bladder tissue; the expression level was greater in higher grade and advanced-stage cancer.
Matsuyama M, 2009 [66]	tissue specimens from 30 patients with testicular cancer and 10 patients with normal testes	Strong CysLT_1_R expression was observed in testicular cancer specimens, in contrast to weak expression in normal testes.
Matsuyama M, 2007 [67]	Tumor specimens were obtained from patients with prostate cancer (*n* = 151), prostatic intraepithelial neoplasia (*n* = 20), benign prostatic hyperplasia (*n* = 20), and normal prostate (*n* = 20)	Prostate cancer tissue had a significantly more extensive and intense CysLT_1_R expression, in nuclei and cytoplasm, than other groups, and the expression was stronger in those with higher Gleason score.
Funao K, 2008 [68]	Specimens from 58 patients with renal cell carcinoma and 20 patients with normal kidney tissues	Significantly more extensive and intense CysLT_1_R expression, in nuclei and cytoplasm, was observed in renal cell carcinoma tissues, compared with normal kidney tissue; the expression of CysLT_1_R was stronger in cancer tissues with higher grade.
Breast cancer		
Magnusson C, 2011 [74]	specimens from 144 breast cancer patients	Breast cancers with higher CysLT_1_R and lower CysLT_2_R expression levels were associated with higher histological grade and worse overall survival.
Other malignancies		
Sveinbjörnsson B, 2008 [78]	27 neuroblastoma surgical specimens, 3 childhood ganglioneuromas, 3 samples of nonmalignant adrenals from children	CysLT_1_R staining was evident in all clinical tumor specimens and the adjacent vasculature.
Slater K, 2020 [81]	data of 80 primary uveal melanoma samples in The Cancer Genome Atlas (TCGA) and tissue from 52 patients of primary uveal melanoma in the Liverpool Ocular Oncology Centre	The data from TCGA showed that higher expression of *CYSLTR1* and *CYSLTR2* genes were significantly associated with poorer disease-free survival and overall survival. However, analysis with tissue microarray of 52 patients only showed poorer overall survival in those with higher CysLT_1_R expression but CysLT_2_R expression was not associated with survival.
Chemopreventive effects of cysteinyl leukotriene inhibition		
Tsai MJ, 2016 [105]	4185 CysLTR antagonist (LTRA) users and 20,925 LTRA non-users from Taiwan National Health Insurance Research Database	LTRA use decreased cancer risk in a dose-dependent manner in asthma patients. The chemopreventive effect of LTRA was markedly observed in terms of lung, colorectal, liver and breast cancer.
Sutton SS, 2021 [106]	23,730 patients with leukotriene pathway inhibiting medication exposure and 534,736 patients from the data from the Department of Veteran Affairs	Patients with leukotriene pathway inhibiting medication (montelukast, zafirlukast, or zileuton) exposure had a significantly reduced risk of lung cancer than those without exposure.
